# Foreign Gleanings

**Published:** 1874-07-01

**Authors:** F. J. Huse


### FOREIGN GLEANINGS.

COLLATED BY F. J. HUSE, M.D.

INTRAVENOUS INJECTION OF CHLORAL.—An account has recently been published in the *Journal de Therapeutique*, of a well-marked case of tetanus, in which recovery followed the injection of hydrate of chloral in three doses of fifty grains, at intervals of twenty-four hours. The paralysis of sensibility and mobility, said to have closely resembled surgical shock, was complete, and there was no appearance of phlebitis. Great care, however, is required, if the solution is of sufficient strength to be of any service, to avoid injecting any of it into the surrounding cellular tissue, as it is liable to produce a most painful abscess.

It is especially urged that chloral is incapable of paralyzing the reflex action of the cord except when administered by direct intravenous injection. This method has also been chosen by M. Ore, who claims to have solved the problem of anæsthesia by thus maintaining insensibility for any necessary length of time, and afterwards being able to restore consciousness very speedily by the application of electricity. The injection of five and a half drams of a solution of chloral in three times its weight of water kept a patient in a calm sleep during an operation for necrosis of the astragalus, occupying nearly half an hour.

The further investigation of the phenomena which follow this method of administration has been chosen as the subject of his present course of lectures by M. Vulpian, the professor of experimental pathology, who takes the chair left vacant by the departure of Brown-Sequard from Paris.

---

ENGLISCH ON CYSTS OF THE POSTERIOR WALL OF THE BLADDER IN MAN.—In the *Anzeiger der k.k. Gesellschaft der Aertze in Wien*, January, 1874, Dr. Englisch has a communication on these tumors. If a sufficient number of preparations be examined, there are not unfrequently found, in the space between the two vasa deferentia, and less often on the vesiculæ seminales, cysts of various sizes. He describes them as consisting of four different kinds.

The first class of cysts corresponds with the mesial line, and lies in the muscolo-fibrous membrane, which binds together the vasa deferentia.

The second kind lies more to the side, near the vas deferens, and is connected with it by means of a process of connective tissue.

The third form lies partially in the prostate, and corresponds with an enlarged sinus pocularis, if its orifice be closed.

A fourth form is connected with the vesicula seminalis, and has no attachment to the vas deferens.

These cysts do not only exist in adults, but are also met with in children, and it has been clearly shown that the first three kinds exist in newly-born infants, and become developed later on.

It is probable that the cyst lying in the middle line corresponds with the range of the Mullerian canals, and that those of the second form proceed from the development of the orifice of the vas deferens, or represent the remains of the cul-de-sac of the Wolffian body.

The third form is developed when the cul-de-sac of the sinus pocularis, which commences at the colliculus, extends far backwards, and becomes distended.

The fourth species is found in connection with the results of inflammation of the vesiculæ seminales, so that the closure of a sinus is peculiarly a result of an inflammatory process.

So long as the cysts are small, they cause no particular annoyance, although they may, as in instances proved, distend the recto-vesical pouch and cause retention of urine.

DOUTRELEPONT ON A RARE FORM OF SCIRRHUS OF THE MALE BREAST. Professor Doutrelepont, (*Berliner Medicinische Wochenschrift*, March 14,) relates a case of a rare form of scirrhus of the male breast (squirrhe pustuleux ou dissemine of Velpeau). Carcinoma of the male breast is very uncommon, and this particular form is still more so. The patient was fifty years old, weak and sickly looking. In 1870 he first noticed a hard swelling in his left nipple, which, however, did not cause much pain. It was treated with iodine ointment. In 1872 he first felt pain from the pressure of his braces, and from that time there was a general enlargment of the swelling. In February, 1873, it ulcerated and spread. This was treated with nitrate of silver. In May, when he first came under observation, the mass had become attached to the ribs. The ulcerated surface was somewhat circular, and had a diameter of about $2\frac{3}{4}$ inches. The base was very much excavated, cicatrized at one little spot and very hard. There were several hard knots in the skin, and at the edge of the axilla was a movable mass, as large as a pigeon's egg. At the edge of the sternum and close to the xiphoid process, there were two knots attached to the cartilages, and a good many of the axillary glands were enlarged and hard; between this time and July a dozen new tumors had appeared. The ulcer was treated, after Burow's method, with powdered chlorate of potash.

Two contiguous tumors were removed, and a microscopical examination showed scirrhus. In the proximity of these tumors there was a cell-infiltration through the entire skin in the form of canals, which ran obliquely to the surface, in different places, irregularly dilated, ramifying, and, on closer examination, leaving no doubt that they were in connection with the lymphatics of the skin. Where this cell-infiltration involved the epithelium of the glands of the skin or of the epidermis, great poliferation was manifested. It is probable that the scirrhus in this case spread through the lymphatics of the skin.

DR. LIVINGSTONE ON ALCOHOL.—Dr. Livingstone wrote as follows about sixteen or seventeen years ago: "I have acted on the principle of total abstinence from all alcoholic liquors during more than twenty years. My individual opinion is that the most severe labors or privations may be undergone without alcoholic stimulants." We are informed that the illustrious traveler held to his opinion and practice to the last.—*Medical Press and Circular*.

FELTZ AND RITTER ON AMMONIÆMIA.—The authors record, in the *Comptes Rendus*, experiments from which they deduce that urine in affections of the genito-urinary apparatus is very rarely ammoniacal. In the immense majority of cases of alkalescence, the default is supposed to be in the want of cleanliness of the vessels employed, these containing albuminoid substances more or less changed.

THE GERMAN UNIVERSITIES.—Professor von Recklinghausen, now of Strasburg, has been invited to the chair of Pathological Anatomy in Vienna, in the place of Professor Rokitansky. Dr. Koster, of Giessen, has been chosen Professor of Pathological Anatomy in Bonn, in the room of Professor Rindfleisch, who goes to Wurzburg.

TRICHINOSIS.—In Gossengrun, a small town in Bohemia, sixty persons have been afflicted with trichinosis from eating diseased pork, and six have already died. There have also been recently some cases in the hospital at Pesth.

